# Ectopic Pregnancy in the Cervix: A Case Report

**DOI:** 10.1155/2011/858241

**Published:** 2011-10-26

**Authors:** Mohammad R. Mohebbi, Kurt A. Rosenkrans, Eric E. Luebbert, Tauhni T. Hunt, Michael J. Jung

**Affiliations:** ^1^Department of Family Medicine, Siouxland Medical Education Foundation-University of Iowa, Sioux City, Iowa 51104, USA; ^2^Department of Radiology, St Luke's Regional Medical Center, Sioux City, Iowa 51104, USA; ^3^Siouxland Obstetrics and Gynecology, Sioux City, Iowa 51104, USA

## Abstract

Cervical pregnancy is an extremely rare condition with potential grave consequences if not diagnosed and treated early enough. We present a case and an ultrasound image of early cervical ectopic pregnancy with a history of two previous cesarean sections who was successfully treated with suction curettage.

## 1. Introduction

Cervical ectopic pregnancy is extremely rare, accounting for less than 1% of all ectopic pregnancies [1]. Its etiology is still unclear. However, there are reports of association with chromosomal abnormalities as well as a prior history of procedures that damage the endometrial lining such as cesarean section, intrauterine device, and in vitro fertilization [2].

## 2. Case Report

**Figure 1:**
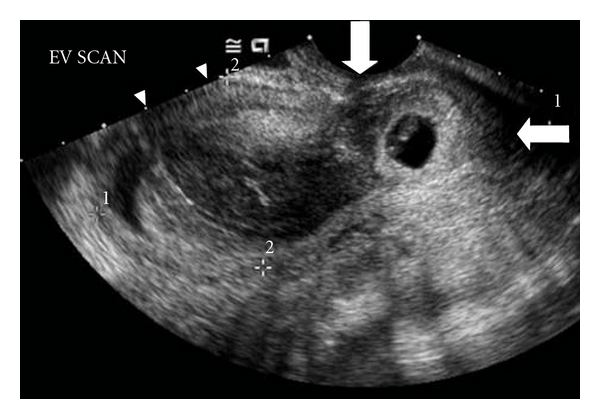
A gestational sac with a small embryonic pole with a fetal heartbeat of 122 bpm located in the cervix below the scar of the previous cesarean section (vertical arrow). Cervix was closed, enlarged, and tender (horizontal arrow). Estimated gestational age based on last menstrual period was 6 weeks and 6 days.

A 25-year-old Caucasian female gravida 4 with a history of two cesarean sections at full term and one spontaneous abortion presented to our clinic with vaginal bleeding. Quantitative beta hCG was 159 mLU/mL. Baseline transvaginal ultrasound showed a well-defined uterus with small amount of fluid in the lower segment of the uterus at the cervical canal with no gestational sac. A repeat transvaginal ultrasound after one week confirmed a gestational sac at the very lower segment of the uterus essentially below the prior cesarean section scars and in the cervix. Fetal heart rate was 101 beats per minute and estimated gestational age was 6 weeks and one day based on the last menstrual period. Quantitative beta hCG was 3490 mLU/mL at this point. Ultrasound was repeated five days later and showed the gestational sac in the cervical canal with a fetal heart rate of 122 beats per minute (Figure 1) and quantitative beta hCG was 10694 mLU/mL. Speculum exam showed a copious amount of bleeding and clots and an enlarged and very tender closed cervix. Microscopic study of the vaginal blood did not show evidence of placenta or fetal tissue. Suction curettage of the gestational sac was performed four days later and the tissue was sent to pathology which confirmed immature placenta and decidual tissue. Beta hCG was 15437 mLU/mL on the day of procedure and 187 mLU/mL and 2 mLU/mL 2 weeks and 2 months after the procedure, respectively. Bleeding stopped completely and the patient resumed her normal periods. Written informed consent was obtained from the patient to publish this case.

## 3. Discussion

In our patient, the fetus was implanted below the previous cesarean section scar. The uterus was empty and the gestational sac showed evidence of fetal heart rate at 6-7 weeks in two different ultrasounds. Cervix was distended on ultrasound exam and there was no sliding of the gestational sac upon applying pressure with the ultrasound probe. Color Doppler also confirmed blood flow around the gestational sac. Unlike true cervical pregnancy, cervical abortion is suggested by the body of the uterus being larger than in the nongravid state owing to the recent loss of the intrauterine sac. Serial ultrasound examinations performed over a few days should distinguish the cervical abortion by the transience of the sac if the diagnosis is in doubt [3, 4]. Two ultrasounds in our patient showed evidence of fetal heart activity and placement of the gestational sac in the cervix. Patients with cervical pregnancy classically present with painless first trimester vaginal bleeding, although several case reports (including ours) describe patients with cramping pain.

Treatment choices may be divided into five categories: tamponade, reduction of blood supply, excision of trophoblastic tissue, intra-amniotic feticide, and systemic chemotherapy [4]. In most reported cases of cervical pregnancy, treatments from more than one category are used [4]. Our patient presented with active bleeding and from the beginning, termination of the pregnancy was strongly favored by both physicians and the patient. 

Treatment with methotrexate chemotherapy of patients with either viable or nonviable cervical pregnancies at <12 weeks' gestation carries a high success rate for preservation of the uterus [5]. Although we considered antimetabolite medications such as methotrexate, studies have shown unsatisfactory results if serum beta hCG is more than 10000 IU/L [6] which was the case in our patient. 

Cervical pregnancy is a rare condition that can be life threatening if not diagnosed and treated early during the course of pregnancy. Increasing trend of cesarean sections and using other invasive methods such as intrauterine device and in vitro fertilization seems to contribute to a higher prevalence of cervical pregnancies these days. This requires that primary care providers who are involved in obstetric care include this entity in the differential diagnosis of women presenting with bleeding and cramping early in pregnancy, as early diagnosis and consult for interventional management is necessary in preserving patient's fertility without significant complications.
